# Harnessing the power within: engineering the microbiome for enhanced gynecologic health

**DOI:** 10.1530/RAF-23-0060

**Published:** 2024-04-15

**Authors:** Caitriona Brennan, Kristina Chan, Tanya Kumar, Erica Maissy, Linda Brubaker, Marisol I Dothard, Jack A Gilbert, Katharine E Gilbert, Amanda L Lewis, Varykina G Thackray, Amir Zarrinpar, Rob Knight

**Affiliations:** 1 Department of Pediatrics, University of California San Diego, La Jolla, California, USA; 2 Division of Biological Sciences, University of California San Diego, La Jolla, California, USA; 3 Department of Bioengineering, University of California, San Diego, La Jolla, California, USA; 4 Medical Scientist Training Program, University of California San Diego, La Jolla, California, USA; 5 Division of Gastroenterology, University of California San Diego, La Jolla, California, USA; 6 Biomedical Sciences Graduate Program, University of California San Diego, La Jolla, California, USA; 7 Department of Obstetrics, Gynecology and Reproductive Sciences, University of California San Diego, La Jolla, California, USA; 8 Center for Microbiome Innovation, University of California San Diego, La Jolla, California, USA; 9 Jennifer Moreno Department of Veterans Affairs Medical Center, La Jolla, California, USA; 10 Institute of Diabetes and Metabolic Health, University of California San Diego, La Jolla, California, USA; 11 Department of Computer Science and Engineering, University of California, San Diego, La Jolla, California, USA; 12 Halıcıoğlu Data Science Institute, University of California San Diego, La Jolla, California, USA

**Keywords:** microbiome manipulation, gynecologic health, polycystic ovary syndrome, bacterial vaginosis, endometriosis

## Abstract

**Graphical abstract:**

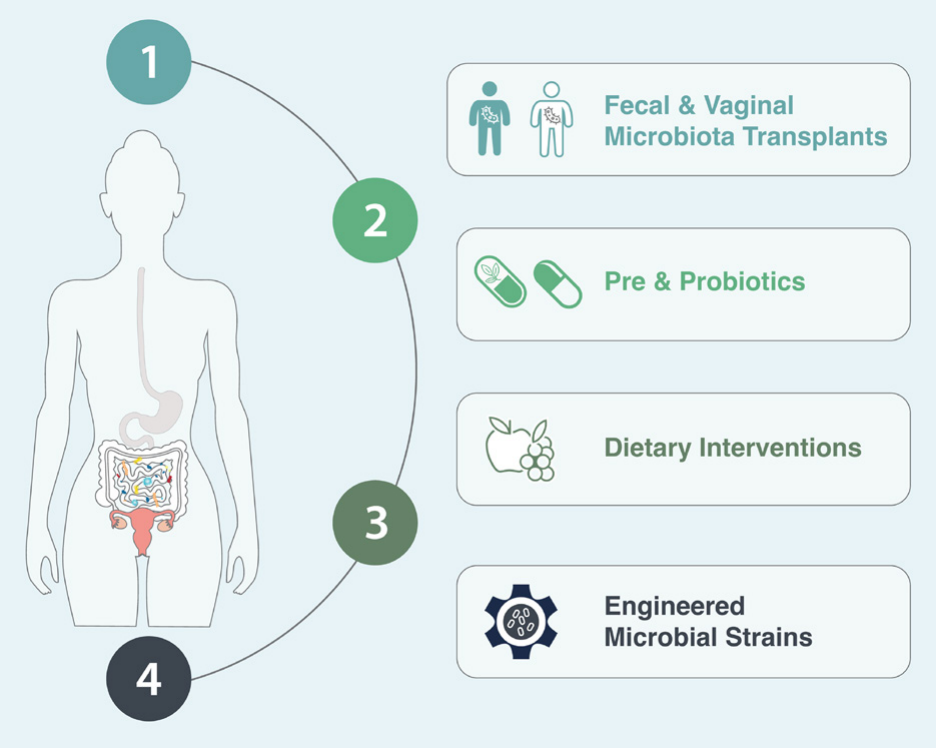

**Abstract:**

Although numerous studies have demonstrated the impact of microbiome manipulation on human health, research on the microbiome’s influence on female health remains relatively limited despite substantial disease burden. In light of this, we present a selected review of clinical trials and preclinical studies targeting both the vaginal and gut microbiomes for the prevention or treatment of various gynecologic conditions. Specifically, we explore studies that leverage microbiota transplants, probiotics, prebiotics, diet modifications, and engineered microbial strains. A healthy vaginal microbiome for females of reproductive age consists of lactic acid-producing bacteria predominantly of the *Lactobacillus* genus, which serves as a protective barrier against pathogens and maintains a balanced ecosystem. The gut microbiota’s production of short-chain fatty acids, metabolism of primary bile acids, and modulation of sex steroid levels have significant implications for the interplay between host and microbes throughout the body, ultimately impacting reproductive health. By harnessing interventions that modulate both the vaginal and gut microbiomes, it becomes possible to not only maintain homeostasis but also mitigate pathological conditions. While the field is still working toward making broad clinical recommendations, the current studies demonstrate that manipulating the microbiome holds great potential for addressing diverse gynecologic conditions.

**Lay summary:**

Manipulating the microbiome has recently entered popular culture, with various diets thought to aid the microbes that live within us. These microbes live in different locations of our body and accordingly help us digest food, modulate our immune system, and influence reproductive health. The role of the microbes living in and influencing the female reproductive tract remains understudied despite known roles in common conditions such as vulvovaginal candidiasis (affecting 75% of females in their lifetime), bacterial vaginosis (25% of females in their lifetime), cervical HPV infection (80% of females in their lifetime), endometriosis (6–10% of females of reproductive age), and polycystic ovary syndrome (10–12% of females of reproductive age). Here, we review four different approaches used to manipulate the female reproductive tract and gastrointestinal system microbiomes: microbiota transplants, probiotics, prebiotics, and dietary interventions, and the use of engineered microbial strains. In doing so, we aim to stimulate discussion on new ways to understand and treat female reproductive health conditions.

## Introduction

The human microbiome comprises trillions of bacteria, archaea, fungi, protists, and viruses that play crucial roles in maintaining health and influencing progression of disease. There is considerable variation in the microbial composition associated with different body sites (Costello *et al.* 2009, The Human Microbiome Project Consortium 2012), and many of these associations, particularly in the vagina, are linked to various gynecologic diseases. An individual's response to a particular type of therapy can be influenced by variations in the composition and functional potential of the microbiome both between people (McDonald *et al.* 2018) and across body sites (Walsh *et al.* 2018). This points to the possibility of developing optimized individual treatment plans through manipulation of the microbial community in a given body site.

Transformation of the microbiome from disease-promoting to health-promoting is a promising avenue to combat diverse human ailments. For example, one of the most well-documented clinically beneficial microbial manipulation approach is fecal microbiota transplantation (FMT) as a therapy for recurrent *Clostridioides difficile* infection (Rohlke & Stollman 2012), in which healthy colonic flora is restored and can outcompete the pathogenic *C. difficile*. Taking it a step further, the live bacterial communities from the human fecal matter of qualified healthy individuals were isolated and approved by the U.S. FDA for oral use in April 2023, making this the second FDA-approved microbiome-based therapeutic (FDA Approves First Orally Administered Fecal Microbiota Product for the Prevention of Recurrence of *Clostridioides difficile* Infection, 2023). In addition to FMT, other more accessible strategies to change the microbiome are currently under investigation, such as probiotic treatments (Kim *et al.* 2019*b*), and dietary interventions (Zeevi *et al.* 2015), which can alleviate cardiometabolic, immune, and even neurological disorders (Gilbert *et al.* 2018). Another approach currently being developed is the use of engineered bacteria that produce therapeutic compounds within the body (Charbonneau *et al.* 2020).

Despite such remarkable progress, our understanding of microbiome dynamics in female-dominant disorders or reproductive health remains critically understudied (Dothard *et al*. 2023). This disparity likely stems from the disproportionate allocation of funding toward disorders more prevalent in males and neglect of conditions predominant in females despite their substantial disease burden (Mirin 2021). Our review highlights the significant knowledge gap in the role of the vaginal and gut microbiomes in the pathogenesis of gynecological conditions.

**Figure 1:**
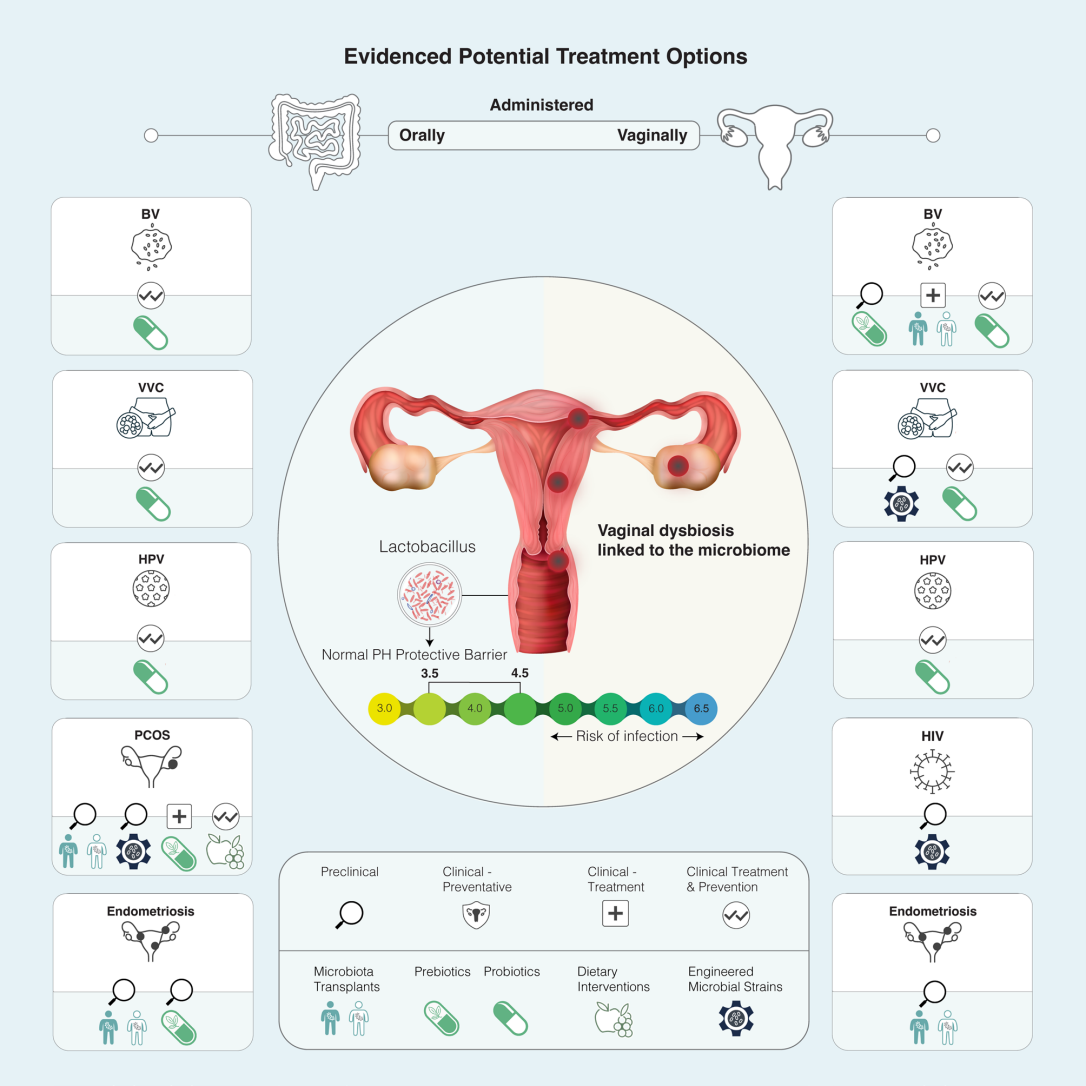
Overview of four strategies designed to support the role of the microbiome in gynecological health. These approaches, as evidenced by clinical trials and preclinical studies, aim to manipulate the microbiome of the gut or vagina to improve gynecologic health and thereby hold the potential for preventing, managing, and treating a range of gynecological issues. 1. Microbiota Transplants: transfer of health-associated bacteria to remediate the vaginal and gut microbiome. 2. Pre and Probiotics: the consumption of beneficial bacteria or specific nutritional components to maintain and promote health. 3. Diet:support of beneficial microbes through dietary components. 4. Engineered Microbial Strains: designing genetically engineered bacteria to achieve health outcomes.

The vaginal and gut microbiota maintain discrete microbial ecologies based on abiotic and biotic factors such as pH, oxygen levels, nutrient availability, differences in epithelial cell structures, and immune surveillance, which, when disturbed, can result in organ-specific disease. The vaginal microbiota’s community composition has been classified into five unique community state types (CSTs), several of which are dominated by lactic acid-producing bacteria belonging to the *Lactobacillus* genus (Ravel *et al.* 2011, Kwon & Lee 2022). These bacteria help create an acidic environment, maintaining the vaginal pH between 3.5 and 4.5, a key protective barrier against pathogenic microorganisms (Fig. 1). This limits bacterial overgrowth linked with bacterial vaginosis (BV) and prevents pathogen colonization linked to cervical cancer (Lewis *et al*. 2017). However, CST-IV and subsequent CST subgroupings are dominated by anaerobic and microaerophilic bacteria, generally thought to comprise a non-optimal microbiome. Notably, many of the seminal studies delineating these groupings are not racially representative, and moving forward it is critical to be racially equitable when conducting such studies to avoid clinical disadvantages (Ravel *et al.* 2011).

While the vaginal microbiome is becoming increasingly characterized, the microbiota associated with the upper female reproductive tract, including the uterine cavity, fallopian tubes, and ovaries, remains severely understudied. However, recent studies have indicated that the uterus is not sterile and instead may be colonized by a low abundance of microorganisms (Moreno *et al.* 2016, Chen *et al.* 2017). This highlights the need for rigorous, validated techniques for transvaginal characterization of the upper reproductive tract microbiome to remove vaginal microbiome contamination (Kennedy *et al.* 2023).

While the gut microbiome is a distinct microbiome site from the female reproductive tract, it displays sexual dimorphism (Sisk-Hackworth *et al*. 2023). When studied through this lens, it is referred to as the ‘microgenderome’ (Flak *et al*. 2013) or, more accurately, the ‘microsexome’ (Mulak *et al*. 2022). The female gut microbiome is associated with sex differences in immunity, disease prevalence, and female reproductive physiology (Yoon & Kim 2021). For example, glucuronide-conjugated estrogen and phytoestrogen can be deconjugated by bacterial β-glucuronidase, which may influence circulating levels of these sex hormones, disrupting steroid receptor-mediated physiological processes distal to the gut, with impacts on reproductive health and menopause (Baker *et al*. 2017, Dothard *et al*. 2023). Thus, sex differences in the microbiome are crucial to incorporate into studies of human health and disease.

This literature review focuses on pre-clinical studies and clinical trials targeting the vaginal and gut microbiomes for the prevention or treatment of prevalent gynecologic conditions. Carefully selected for their potential impact, these studies demonstrate the potential of manipulating the microbiome for enhanced health, encouraging further exploration and research in this promising field. The studies we have chosen are illustrative rather than comprehensive and focus on four main methods of engineering the microbiome for gynecological health: transplant, probiotics, prebiotics and diet, and synthetically designed microbial strains (Fig. 1). Finally, we strive to use gender-inclusive language, and our use of the word ‘female’ refers to individuals assigned female sex at birth. We also explore the role of the vaginal microbiome in gender-diverse individuals, specifically transgender men and transgender women.

## Microbiome-linked gynecological diseases discussed

Before delving into methods of microbial manipulation, it is critical to understand the diseases for which they are currently under investigation. Here, we briefly review these diseases and their links to the microbiome.

### Bacterial vaginosis

One of the most common etiologies of vaginal symptoms is bacterial vaginosis (BV). BV impacts roughly a quarter of females globally, with some variation by region and ethnic background (Peebles *et al.* 2019). It is a non-STI biofilm-based disease arising from vaginal dysbiosis classically associated with fewer Lactobacilli present in the vagina and an increase in anaerobes, such as *Gardnerella vaginalis*,* Fannyhessea* (previously *Atopobium*)* vaginae*, and *Mycoplasma genitalium* (a few of the ‘BV-associated bacteria’). Lactic acid-producing Lactobacilli (*Lactobacillus acidophilus, Lactobacillus crispatus, Lactobacillus jensenii,* and *Lactobacillus gasseri*) are dominant in the non-BV state and out-compete anaerobic bacterial adherence to the vaginal epithelium, thus preventing BV (Pramanick *et al.* 2019, He *et al.* 2020*a*). Additionally, BV is linked with an increased risk of contracting STIs, including HIV (Brotman 2011, Abbai *et al*. 2016, Armstrong *et al.* 2022). Understanding BV-associated microbial disruption in humans is challenging due to the differences in the vaginal microbiome profile in individuals of different ethnic groups, with diet and lifestyle implicated in these differences (Ravel *et al.* 2011). The current standard of care is antibiotics against anaerobic bacteria: either oral or intravaginal metronidazole or intravaginal clindamycin is prescribed. There have been no new antibiotic treatments for BV in the past 50 years, and with post-treatment relapse rates reported up to 80%, it is imperative to consider novel therapeutic approaches (Abbe & Mitchell 2023).

### Vulvovaginal candidiasis

Vulvovaginal candidiasis (VVC, ‘yeast infection’) is commonly caused by overgrowth of the opportunistic yeast *Candida albicans*. Approximately 75% of females experience at least one yeast infection in their lifetime (Vaginal yeast infection (thrush): Overview, 2019), with 8% developing recurrent yeast infections after treatment. Growing resistance to treatment drugs is on the rise (Oerlemans *et al.* 2020, Jeanmonod & Jeanmonod 2023). The current standard of care uses ‘-azole’ drugs, which impair yeast cell wall integrity. However, recurrence after administration of oral antibiotics for a different primary concern is common and may be related to an antibiotic-induced reduction of beneficial gut microbes, thus producing a non-optimal vaginal microbiome (Falagas 2006). Lactobacilli are thought to modulate the entire vaginal microbial community, and the cell-free supernatant of *L. crispatus, L. jensenii,* and* L. gasseri* significantly reduce *C. albicans* growth *in vitro* (Wang *et al.* 2017). One proposed mechanism is competition for adherence to the vaginal epithelium (Boris *et al.* 1998, Oerlemans *et al.* 2020). Given the intricate relationship between VVC and the host microbiome, manipulating the microbiome to prevent and treat VVC is a logical next step.

### Human papillomavirus

Another common reproductive disease associated with the microbiome is infection with Human Papillomavirus (HPV), which is recognized as the causative form of cervical cancer. While certain forms of HPV are carcinogenic, HPV can be cleared spontaneously by 90% of females, and the microbial dynamics leading to HPV clearance are a topic of active investigation (Veldhuijzen *et al.* 2010). Non-optimal vaginal microbiomes are associated with an increased risk of HPV infection (Lehtoranta *et al.* 2022), positing that probiotic interventions could help prevent HPV infection and persistence. The BV-associated bacteria *Gardnerella* and *Fannyhessea* are commonly associated with HPV infection (Wei *et al.* 2021, Yang *et al.* 2022*a*). In fact, Lactobacillus-depleted microbiomes or those with predominantly BV-associated bacteria have higher rates of HPV persistence (Di Paola *et al.* 2017). Conversely, certain microbes are associated with quicker HPV clearance; *L. gasseri*-dominated microbiomes seem to have faster clearance rates than microbiomes with low lactobacilli and high *Fannyhessea* (Brotman *et al.* 2014). It is notable that *Lactobacillus iners* is a common member of the vaginal microbiome in individuals regardless of HPV carrier status. However, *L. iners*-dominated microbiomes (with decreased *L. crispatus* and *L. gasseri*) appear to have poorer outcomes and are associated with VVC (Verstraelen *et al.* 2009, Audirac-Chalifour *et al.* 2016, Jang *et al.* 2019, Sabbatini *et al.* 2021). Nonetheless, there appears to be a ‘chicken and egg’ paradox between HPV and the vaginal microbiome residents, with one appearing to shape the other (Lebeau *et al.* 2022).

### Endometriosis

Endometriosis is characterized by the translocation and growth of endometrial tissue outside of the uterus, causing a chronic inflammatory response. This debilitating condition affects approximately 6–10% of females of reproductive age (Saunders & Horne 2021, Stephens *et al.* 2022). Endometriosis is a common cause of chronic pelvic pain, can cause infertility and dysmenorrhea, and is a risk factor for ovarian cancer (Kok *et al.* 2015). However, it is not easily diagnosed as surgical exploration is the gold standard. There is currently no known cure for endometriosis, and treatment aims to control symptoms. Current treatments include surgical removal of lesions and hormone-suppressive therapy (Saunders & Horne 2021, Stephens *et al.* 2022). There is a large unmet need for robust therapeutics and non-invasive biomarkers for diagnosis. Endometriosis pathogenesis is associated with compositional changes in the microbiota of both the reproductive tract and the gut (Khan *et al.* 2014, 2016, Chen *et al.* 2017, Yuan *et al.* 2018, Akiyama *et al.* 2019, Ata *et al.* 2019, Wessels *et al.* 2021). For example, 64% of a cohort of 155 participants with endometriosis had *Fusobacterium* in the endometrium, while only 7% of participants without endometriosis were *Fusobacterium* positive (Muraoka *et al.* 2023). Additionally, microbial metabolites such as short-chain fatty acids are becoming increasingly implicated in the progression of endometriosis (Le *et al.* 2021, Chadchan *et al.* 2023). However, many of these studies are quite recent and do not explore the microbial changes after surgical or hormonal treatment. While these initial studies are quite promising, more work is needed to understand the endometriosis-associated microbiome pre- and post-treatment.

### Polycystic ovary syndrome

Polycystic ovary syndrome (PCOS) is a complex endocrine disease affecting roughly 6–10% of individuals with ovaries, primarily during reproductive years (Bozdag *et al.* 2016). It is diagnosed using the Rotterdam criteria, requiring two out of the following three features: clinical and/or biochemical hyperandrogenism, oligo- or anovulation, and polycystic ovarian morphology (‘Revised 2003 consensus on diagnostic criteria and long-term health risks related to polycystic ovary syndrome’, 2004). PCOS significantly affects physical and emotional well-being, resulting in infertility, menstrual irregularities, acne, excessive male-pattern hair growth, and an increased risk of anxiety and depression. Over 80% of individuals with PCOS also have metabolic dysfunction including insulin resistance (IR) with or without obesity, which can lead to an increased risk of type 2 diabetes, gestational diabetes, cardiovascular disease, and non-alcoholic fatty liver disease (Sanchez-Garrido & Tena-Sempere 2020). While a connection exists between higher body mass index (BMI) and PCOS, it is not a direct cause-and-effect relationship. Elevated BMI, particularly in cases of obesity, can contribute to IR. This, in turn, disrupts hormone levels, potentially worsening PCOS symptoms. Notably, not all women with PCOS exhibit a high BMI, and conversely, not all women with a high BMI develop PCOS (Sam 2007). While the etiology of PCOS is unclear, substantial evidence demonstrating the importance of the gut microbiome in shaping glucose homeostasis and driving metabolic disorders has led to the hypothesis that alterations in the microbiome are also involved in the pathology of PCOS (Giampaolino *et al.* 2021, Rizk & Thackray 2021). While there is inconsistency in the specific gut bacteria reported to be altered in PCOS, a recent meta-analysis of 17 studies showed that there is a consistent decrease in alpha diversity (Sola-Leyva *et al.* 2023), indicating that reduced microbial biodiversity may be another hallmark of PCOS. Furthermore, recent studies are beginning to consider the effect of hyperandrogenism on the vaginal microbiome, where significant differences are observed in vaginal bacteria between individuals with and without PCOS (Hong *et al.* 2020). However, similar to HPV, PCOS also presents a ‘chicken and egg’ paradox, with the interplay between host biology and disease shaping one another.

### Human immunodeficiency virus

Human immunodeficiency virus (HIV) affects an estimated 39.0 million people globally, with females comprising 53% of those living with the virus (Global HIV & AIDS statistics – Fact sheet, 2023). HIV is primarily transmitted through unprotected sexual intercourse, sharing needles, and from birthing parent to child during childbirth or breastfeeding. Untreated HIV can progress to acquired immunodeficiency syndrome (AIDS), in which the immune system becomes severely compromised, leading to increased susceptibility to opportunistic infections and certain cancers. The virus can be present in vaginal and cervical fluids, as well as menstrual blood. A disrupted vaginal microbiome, such as the one found in BV, can increase inflammation and create an environment more conducive to viral transmission, highlighting the potential for vaginal microbiome optimization to reduce HIV transmission (Armstrong *et al*. 2023). Despite advances in effective antiretroviral therapy and pre-exposure prophylaxis (PrEP), HIV remains a major worldwide health concern due, in part, to the barriers that exist to testing and treatment access in many low- and middle-income countries.

## Microbiota transplants

Microbiota transplants transfer microbes from a carefully screened healthy phenotype donor into a like-body site of a recipient with a diseased phenotype. Microbiota transplants can be used both as a beneficial therapeutic and an experimental tool for exploring pathology in animal models. Preclinical animal experiments and clinical trials using both FMTs and vaginal microbiota transplant trials (VMTs) show promising results in female reproductive health. Regulation of microbiota transplants by the FDA is a major consideration due to the risk of infection as a side effect of transplantation, especially in immunocompromised patients. Microbiota screenings for opportunistic pathogens, infectious diseases, and multi-drug-resistant organisms can support microbiota transplant safety and efficacy (Carlson 2020, Yockey *et al.* 2022) for both autologous and nonautologous donors. However, historical barriers to microbiota transplantation may hinder access to patients, specifically due to the sourcing of screened donor material, logistic challenges of delivering the resulting fresh treatment preparations, and expenses associated with pathogen screening (Panchal *et al.* 2018, Kim *et al.* 2019*a*).

### Fecal microbiota transplant

FMTs entail the transfer of fecal microbes from a healthy donor to a recipient with a non-optimal microbiome. Multiple studies have demonstrated the therapeutic potential of FMTs in PCOS (Guo *et al.* 2016, Yang *et al.* 2022*b*). For instance, letrozole-induced rat models of PCOS show improvement in estrous cyclicity after FMT from healthy rats or treatment with a probiotic containing *Lactobacillus* (Guo *et al.* 2016, Yang *et al.* 2022*b*). Moreover, these results also implicate microbial disruption as a potential driver in PCOS pathogenesis. While the mechanism of action remains unclear, gut microbiome manipulation using FMT has led to decreased androgen levels and normalized ovarian morphology, further highlighting the influence of the gut microbiome on the female reproductive tract. Clinical studies will help determine whether FMT is a viable treatment option for patients with PCOS. Of note, previous studies demonstrate that a single administration of FMT in chronic metabolic conditions such as obesity and diabetes does not lead to long-term improvements in outcomes (Vrieze *et al.* 2012, Kootte *et al.* 2017). Hence, more frequent or persistent treatment may be necessary to see beneficial results in chronic metabolic conditions such as PCOS (Baunwall *et al.* 2020).

In addition to the therapeutic application of healthy FMT, investigators can use the transplantation of disease-associated FMT as a tool to provide a deeper understanding of the underlying pathology of various gynecological diseases. For example, transplantation of disease-associated microbiota can trigger pathology, including PCOS-like phenotypes in rodents (Qi *et al.* 2020, Han *et al.* 2021, Yang *et al.* 2022*a*), ovarian tumor development in mice (Wang *et al.* 2022), and endometriosis disease progression in mice (Chadchan *et al.* 2023). Such findings support the idea that the gut microbiome contributes to the progression or prevention of gynecological diseases.

Future studies can also focus on the potential for autologous FMT (aFMT), whereby a patient is both the donor and recipient (Suez *et al.* 2018). Autologous FMT consists of banking the host’s microbiome during a healthy state and later transplanting it to the same host when diseased, thus potentially improving long-term sustainability. If aFMT proves to be a successful intervention for gynecological diseases, it would require either better predictors of who will develop disease or more accessible stool banking opportunities. Given that FMT may work for a number of medical ailments, aFMT may have broad appeal.

### Vaginal microbiota transplant

The success of FMTs has laid the groundwork for transplantation of other microbiota sites, such as the vagina. Vaginal microbiota transplants (VMTs) have also shown considerable results in improving certain gynecological outcomes. VMT has been primarily investigated in BV, with the seminal study following five patients treated with VMT one week post intravaginal antibiotic treatment (Lev-Sagie *et al.* 2019). Four out of five showed marked symptom improvement and a shift to a remediated, *Lactobacillus*-dominated vaginal microbiome.

Additionally, preclinical experiments in animal models with VMT have revealed a more discrete and modular understanding of the causal factors of BV. However, it is important to note that the reproductive tract, physiology, timing of estrous, and microbiome of animals are considerably different from those of humans, and also vary between different animal models (Noguchi *et al*. 2003, Guo *et al.* 2016). Despite these caveats, we can still derive mechanistic understanding using these models. For example, one group made a rudimentary mouse model of BV using eight successive days of vaginal inoculation with high levels of *G. vaginalis* (Li *et al.* 2023*a*). Both VMT and a synthetic bacterial consortia transplantation (comprised of isolates of *L. crispatus*, *Lactobacillus rhamnosus*, *Lactobacillus salivarius*, and *Lactobacillus plantarum* from vaginal discharge of healthy females) rescued the diseased phenotype (Li *et al.* 2023b), with the VMT more effective at suppressing inflammation. However, a major caveat of this study is that there are likely considerable differences between the human BV biofilm and this *G. vaginalis*-induced mouse model. Nonetheless, it is an encouraging first step in establishing a model for BV, which may ultimately help pave the way for the first FDA-approved VMT.

VMT is also used in animal models to explore gynecological pathology, with one study transferring human vaginal lavage fluids from ten females with endometriosis, ten females with BV, and ten healthy females into the vaginas of healthy rats (Wang *et al.* 2021*a*). This led to significantly higher uterine inflammation in the endometriosis group as compared to the healthy and placebo controls. The endometriosis and BV lavage recipient rats showed epithelial lesions consistent with inflammation in the endometrial tissue. This study is a step toward untangling the complicated microbial dynamics that contribute to BV and endometriosis, as Koch’s postulates appear to be partially fulfilled. Thus, VMTs have potential use both in clinical treatment and in discovery-based research on disease etiology making them one a highly promising method of engineering the female reproductive microbiome.

## Probiotics

Probiotics, as defined by the International Scientific Association of Probiotics and Prebiotics, are ‘live microorganisms that, when administered in adequate amounts, confer health benefits on the host’ (Hill *et al.* 2014). Probiotics, unless claimed to help treat a disease, are not regulated by the FDA, and their usage dates back many generations (McFarland 2015). As we progress in our understanding of potentially beneficial microbes, it is critical to be cognizant of the contexts in which they are efficacious, rather than using the term ‘probiotics’ as a panacea. Using probiotics for reproductive health has led to potentially promising results, which we will explore in-depth using three conditions: BV, VVC, and HPV infection. We have included studies exploring both oral and vaginal probiotic administration. Although the precise route of oral probiotics to the vagina via the gastrointestinal tract is not fully understood, it is crucial to note that probiotics do not necessarily need direct access to the vagina to affect the reproductive tract microbiome (Borges *et al*. 2014). Furthermore, the indirect mechanisms through which orally administered probiotics influence the vaginal microbiome remain to be fully elucidated.

In a 2020 study, investigators assessed the efficacy of LACTIN-V, a strain of *L. crispatus*, in 152 premenopausal participants aged 18–45 years with recurrent BV (Cohen *et al.* 2020). After metronidazole treatment, LACTIN-V was administered intravaginally daily for 5 days and then twice weekly for 10 weeks. At 12 weeks post-treatment, 30% of LACTIN-V users relapsed vs 45% in the placebo group. However, at 24 weeks, the groups had similar relapse rates (12% LACTIN-V vs 17% placebo), and the amount of vaginal LACTIN-V decreased over time, most likely highlighting the transience of probiotics. Nevertheless, LACTIN-V usage was also associated with decreased inflammatory markers (Armstrong *et al.* 2022).

The efficacy of probiotics may be dictated by the strain and the dosing regimen. For example, one report showed that the intermittent use of a probiotic with various strains of *Lactobacillus* and *Bifidobacterium* for 2 months was useful in treating BV with similar efficacy to oral metronidazole and better than no treatment (Van De Wijgert *et al.* 2020). They reported no significant therapeutic effect of an intermittent 2-month use of a probiotic with *L. rhamnosus*. In contrast, Reid *et al.* (2003), reported that daily use of oral *L. rhamnosus* GR-1 and *Lactobacillus fermentum* RC-14 for 60 days showed significant improvements in the microbial composition of patients with asymptomatic BV (Reid *et al.* 2003). Thus, perhaps adherence to daily vs intermittent regimen timing may be a driving factor in the usefulness of probiotics.

The importance of when probiotics are administered is further highlighted by a study reporting lower relapse rates when administering probiotics directly after menstruation. Larsson *et al.* (2008) administered daily clindamycin treatment for 7 days, followed directly by vaginal administration of *L. gasseri* and *L. rhamnosus* for 10-day blocks over the course of 4 months (Larsson *et al.* 2008). At the end of the study (6 months), 35% of participants on probiotics relapsed compared to 54% of participants on a placebo pill. Furthermore, the probiotic treatment group relapsed significantly later than the placebo group. Menstruation is important to consider because there appears to be a higher concentration of non-*Lactobacillus* species during menstruation when menstrual blood also raises the vaginal pH, which may contribute to compositional instability (Eschenbach *et al.* 2000). Further research addressing the timing of treatment in relation to the menstrual cycle is needed.

Similar to BV, the idea of recolonizing the vagina for protection against vulvovaginal candidiasis (VVC, ‘yeast infection’) has been discussed for generations (Wood *et al.* 1985). Clinical VVC trials using Lactobacilli have had varying efficacy, and there is more research needed before making strong clinical recommendations. Oerlemans *et al.* conducted a trial with a vaginal gel comprising *L. rhamnosus*, *L. plantarum*, and *L. pentosus* used once daily for 10 days (Oerlemans *et al.* 2020). There was little benefit compared to fluconazole usage, with 55% of participants not responding to the gel alone and requiring fluconazole therapy. However, participants who responded to the probiotic gel had a similar fungal burden as those on antifungal fluconazole therapy at 4 weeks. Similarly, in a separate study, investigators observed yeast depletion at 4 weeks using an oral capsule of *L. rhamnosus GR-1 and L. fermentum RC-14* (Reid *et al.* 2003). However, the gel trial found that fluconazole reduced the number of Lactobacilli, which are thought to be beneficial in protecting against VVC, suggesting that further studies of dual therapy with fluconazole and Lactobacilli may be warranted for a more effective treatment.

In fact, others have investigated such dual therapy. A randomized control trial with fluconazole usage +/− probiotic capsules containing *L. rhamnosus* GR-1 and *L. reuteri* RC-14 (Martinez *et al.* 2009) demonstrated potential clinical efficacy. At 4 weeks, participants taking probiotics and fluconazole had significantly less discharge compared to the placebo pill and fluconazole group. Those on probiotics had significantly less culturable yeast. These findings suggest a beneficial role for Lactobacilli in dual therapy, while also showcasing the necessity to standardize endpoint measurement techniques (Zhou *et al.* 2009, Macklaim *et al.* 2015).

Finally, while most literature surrounding probiotics discusses bacteria, it is important to be cognizant of other potentially beneficial microbes such as fungi. One study in particular, using a mouse model of VVC, showed promising results using both live and inactivated *Saccharomyces cerevisiae*. By day 4 of probiotic administration, there were comparable results to fluconazole usage. In particular, the live yeast aided in accelerated pathogen clearance (Pericolini *et al.* 2017).

The efficacy of probiotics has also been investigated in cervical infection with high-risk HPV, the primary cause of cervical cancer (Ou *et al.* 2019). Studies of probiotics for the prevention or treatment of cervical HPV infection have had variable success. A 2013 study found that people with precancerous cervical lesions were twice as likely to clear any cytological abnormalities when drinking Yakult, which contains *L. casei* Shirota, for 60 days (Verhoeven *et al.* 2013). In contrast, other investigators reported no difference in high-risk HPV clearance in patients taking a daily oral pill with *L. rhamnosus* GR-1 and *L. reuteri* RC-14 (Ou *et al.* 2019). In addition to the studies testing different strains, Yakult has roughly 20 billion CFUs whereas the pill with *L. rhamnosus* GR-1 and *L. reuteri* RC-14 5.4 billion CFUs (*YAKULT Product Information*, no date). Thus, the dosage and/or species may lead to varying results.

In a study following co-infection between cervical HPV and yeast or BV, investigators reported that taking a vaginal *L. rhamnosus* supplement for 6 months along with initial treatment for the yeast or BV was associated with a twice higher chance of clearing the HPV as compared to those taking the probiotic for 3 months. Unfortunately, this study did not have a control group without probiotics, which would have been helpful in examining the impact of medication-driven management of dysbiosis (Palma *et al.* 2018). The tablets used in that study had 10,000 CFU/tablet rather than the billions found in the previous two studies, ultimately leading to a total dose that may be permissible due to direct vaginal administration.

Probiotics, particularly Lactobacillus and Bifidobacterium strains, show promise in alleviating symptoms of PCOS in both women and several different mouse models (Guo *et al.* 2016, Zhang *et al.* 2019*a*,*b*, He *et al.* 2020*b*). Another study suggested that co-supplementation of probiotics with vitamin D improves mental health, testosterone levels, and hirsutism in women with PCOS (Ostadmohammadi *et al.* 2019). Probiotics alone and synbiotics (co-supplementation of probiotics with prebiotics including resistant dextrin and inulin) improved some clinical markers of PCOS such as free androgen index and sex hormone-binding globulin but had no effect on others, including testosterone and hirsutism (Shamasbi *et al*. 2020). Meta-analyses also indicate positive effects on certain markers of insulin sensitivity and lipid profiles, however, there are no significant changes in other metrics of glycemia and body weight (Liao *et al.* 2018). Probiotics are a promising avenue of clinical symptom management for PCOS; however, further research is needed to fully optimize these interventions.

There are various other important health conditions that have shown to be responsive to probiotics, such as urinary tract infections (UTI), PCOS, ovarian cancer, and Group B *Streptococcus* (the leading cause of neonatal bacterial meningitis), which we urge the reader to further explore (Hanson *et al.* 2022, YulingLi *et al.* 2023). Notably, direct manipulation of the vaginal microbiome using *L. crispatus* has been shown to improve UTI outcomes, pointing to potential microbial cross talk within the urogenital tract (Hanson *et al.* 2022). Thus, while still an active area of research, probiotics provide a potentially exciting and accessible avenue to engineer the vaginal microbiome. In particular, dose, strain, and timing of administration seem to be key effectors.

## Diet and prebiotics

Given the association between poor diet and a non-optimal microbiome (Martinez *et al.* 2021, Makarova & Zyriax 2023), there may be significant therapeutic utility in using diet to alter microbial composition within and beyond the gut. High-fiber foods, particularly those rich in soluble fiber, are broken down by enteric bacteria through a process called fermentation, producing short-chain fatty acids (SCFAs) such as acetate, propionate, and butyrate. SCFAs have several beneficial effects on the body, including providing a source of energy for the cells lining the colon, promoting a healthy gut environment, and potentially reducing inflammation (Caetano & Castelucci 2022, Duan *et al.* 2023). The fermentation of fiber in the gut is governed by several factors including microbial composition, pH, fiber type, and time. A diverse array of gut bacteria enhances the range of fibers that can be fermented, enteric bacteria have optimal pH ranges for fermentation, and the longer the fiber remains in the gut, the more opportunity it is for bacteria to ferment it (Cronin *et al.* 2021). The metabolic by-products or bioactive compounds produced by probiotic microorganisms during their fermentation process are known as postbiotics and can be taken in supplement form. Furthermore, introduction of prebiotics to the diet, which can select for specific gut microbial species, can help facilitate a health-promoting gut microbial metabolism; for example, fiber supplements can promote the synthesis of SCFAs (Deehan *et al.* 2020). Prebiotics, such as fructooligosaccharides (FOS), galactooligosaccharides (GOS), inulin and lactulose, have shown improved metabolic markers and immunomodulation potentially by stimulating the growth of beneficial bacteria like *Bifidobacterium* and *Lactobacillus* (Fernandes *et al.* 2017, Enam & Mansell 2019, Deehan *et al.* 2020).

In a mouse model of PCOS, a study showed that a 21-day treatment with 0.05 g inulin/100 g body weight led to a reduction in the number of cystic follicles and corpora lutea, along with improvements in inflammatory cytokine levels and insulin sensitivity (Xue *et al.* 2019). These positive effects were attributed to the increased abundance of *Bacteroides* and *Bifidobacterium* in the gut, with *Bifidobacterium* showing strong anti-inflammatory properties. These findings highlight the potential role of inulin in therapy, achieved via gut microbiota modulation. Furthermore, prebiotics can be combined with probiotics to work synergistically to enhance the health-promoting effects on the host. This combination, known as synbiotics, aims to support the survival and activity of probiotics by providing them with a favorable environment for growth and colonization. One clinical trial demonstrated that dietary administration of a synbiotic supplementation of fructooligosaccharides, inulin, and various Bifidobacterium and Lactobacillus species significantly reduced testosterone and BMI, both factors linked to PCOS symptoms. By targeting these factors simultaneously, such interventions hold the potential to alleviate various facets of PCOS, including improving hormonal balance and enhancing metabolic function. This approach proved significantly more effective than relying solely on lifestyle and dietary modification (Chudzicka-Strugała *et al.* 2021). Nonetheless, further investigation is needed to understand the specific mechanisms of gut bacteria in PCOS and related metabolic disorders.

Although clinical guidelines do not currently specify a particular diet for optimal PCOS management (Moran *et al.* 2020), dietary interventions have the potential to mitigate hyperandrogenism, obesity, and insulin resistance. In a review that pooled data from 20 RCTs involving 1113 participants, Shang *et al.* reported the maximized benefits of Mediterranean and low-carbohydrate diets for optimizing fertility outcomes, and calorie restriction for ameliorating hyperandrogenism (Shang *et al.* 2021). An additional study analyzed 14 individuals with PCOS that received a high-fiber diet composed of whole grains, traditional Chinese medicinal foods, and prebiotics (WTP diet) for 12 weeks (Wang *et al.* 2021*b*). Adherence to the diet resulted in the alleviation of PCOS clinical phenotypes such as the inflammatory state, lower BMI, decreased levels of leptin (a brain–gut hormone that dictates satiety), and fasting plasma insulin. However, those on the WTP diet also displayed higher testosterone levels and, after an initial dip at 4 weeks, higher fasting blood glucose levels. Given these conflicting outcomes, a small sample size, and a relatively short timeline, further research into the WTP is warranted. Nonetheless, this experiment explored the interesting question of whether fiber intake can mitigate PCOS symptoms. This was also investigated in a case–control study that demonstrated an inverse correlation between dietary fiber consumption, obesity, and insulin resistance among individuals with PCOS and BMI-matched controls (Cunha *et al.* 2019). Additionally, a 3-month intervention of starch resistant to digestion in the small intestine (wheat/corn dextrin; 20 g/day) compared to an insoluble fiber control in people with PCOS demonstrated a significant improvement in testosterone, fasting blood glucose, total cholesterol, LDL-C, HDL-C, triglyceride, and hsCRP (a marker of inflammation) (Gholizadeh Shamasbi *et al.* 2019). These studies indicate that increased dietary fiber or prebiotics supplements may modulate the gut microbiome and consequently improve symptoms of PCOS. Additional studies are needed to determine which dietary fibers and doses are optimal for treating PCOS either alone or in combination with other therapies.

Prebiotics can also be therapeutically beneficial in endometriosis. Chadchan *et al.* investigated the role of gut bacteria and SCFAs in promoting or protecting against the growth of endometriosis lesions (Chadchan *et al.* 2021). SCFAs, as previously mentioned, are by-products of bacterial fermentation and regulate host metabolism. The study found that feces from mice with endometriosis contained less n-butyrate, one of the most abundant SCFAs, in contrast to those without endometriosis. Treatment with n-butyrate reduced the growth of both mouse endometriotic lesions and human endometriotic lesions in a preclinical mouse model, acting in part through G-protein-coupled receptors, GPR43, and GPR109A (Chadchan *et al.* 2021). GPR43 and GPR109A receptor inhibition in an endometrial cell line partially restored cell viability in n-butyrate-treated cells, highlighting potential therapeutic targets.

Certain prebiotics have also been implicated in potentially mitigating BV pathogenesis. One study showed that lactulose promotes the growth of vaginal Lactobacilli in monoculture and in communities cultured from healthy vaginal swabs. Importantly, this promotion did not extend to BV-associated bacteria or *C. albicans* (Collins *et al.* 2018).

Overall, diet and prebiotics have emerged as powerful strategies with potential for improving gynecologic health. By promoting a healthy gut microbiome, hormonal balance, and overall well-being, these interventions offer promising, non-invasive avenues for managing conditions such as PCOS, BV, and endometriosis. Further research is needed to uncover the intricate connections between diet, gut microbiota, and reproductive health, paving the way for personalized interventions and improved outcomes.

## Engineered microbial strains

Engineered probiotics use bacteria that are genetically designed to express a specific function as an alternative to traditional pharmaceutical treatments. Treatment with genetically modified organisms should be considered drug therapy and not, like traditional probiotics, a dietary supplement. The treatment vesicle as a chassis, a bacterial strain that serves as a platform for genetic modification. The choice of chassis is crucial based on manipulability, safety, and scalability, and most studies have selected chassis that are easy to engineer, food-grade, or a predominant species. Recent studies show that there are also some chassis that can be scaled with the use of a prebiotic (Shepherd *et al.* 2018) or using bacteria derived from the system itself, i.e. native bacteria (Russell *et al.* 2022), which can implement a more persistent change. The engineered therapeutic functions themselves include but are not limited to, the production of small molecules, metabolic enzymes, nano-bodies, toxins, and immunomodulators. These engineered probiotics have been designed to treat a range of diseases, including GI disease, infection, dysmetabolism, rare genetic disorders, and cancer (Ma *et al.* 2022, Brevi & Zarrinpar 2023). While studies evaluating individual strains or consortia of bacteria have been somewhat successful in treating vaginal and reproductive conditions, gene-edited probiotics which express deliberate functions are on the horizon for more targeted treatment of these conditions (Vieira-Baptista *et al.* 2022).

For example, several biotechnology companies and academic groups are developing genetically enhanced probiotics against HIV by inserting potent antiviral genes into bacteria that naturally colonize the vagina. Unlike conventional microbicide delivery systems such as gels and films, once administered, engineered probiotics can serve as a more sustained, self-replicating delivery method for the treatment of HIV. The risk of this more sustained treatment method, however, is the persistence of any potentially harmful effects for the patient or the environment and is as of now a risk of unknown impact.

Researchers engineered *L. jensenii* 1153 to produce the potent HIV entry inhibitor cyanovirin-N (CV-N) using chromosomal integration. Pre-clinical testing in macaques showed consistently high levels of colonization with CV-N expressing *L. jensenii* after vaginal administration, with no significant antibody response, and the strain was easily cleared with topical antibiotic administration. In a repeated low-dose challenge model, HIV acquisition was reduced by 63% in macaques, and this engineered bacterium is being explored as a platform to co-express microbicides against HIV and other STIs (Liu *et al.* 2006, Lagenaur *et al.* 2011). More work is needed to examine safety and efficacy in humans.

Furthermore, increased activated genital CD4+ T cells (associated with *L. crispatus* deficiency) and elevated levels of high-risk bacteria indicate a higher HIV risk, initially discovered in a cohort of South African females (Gosmann *et al.* 2017). Investigators are leveraging this discovery to develop targeted engineered bacteria to locally modulate CD4+ expression in the vaginal microenvironment (Gosmann *et al.* 2017). *L. acidophilus* ATCC 4356 engineered to display human CD4 on its surface can adsorb HIV-1 particles by binding to its envelope protein and successfully reduce infection *in vitro* and in a murine model (Wei *et al.* 2019). *Lactobacillus* has also been modulated to express broadly neutralizing nanobodies (VHH) against HIV, which may be a promising immunization method for females at high risk of HIV-1 transmission (Kalusche *et al.* 2020).

Engineered strains are also being actively explored as a method to combat *C. albicans*-driven VVC. Investigators engineered a commercial *Saccharomyces boulardii* strain to produce medium-chain fatty acids (MCFAs) with anti-biofilm and anti-hyphal effects *in vivo*. MCFAs also upregulated the expression of virulence-related genes in the strain of *C. albicans*, SC5314. The constitutive production and secretions of MCFAs serve as a proof-of-concept for the potential of probiotic yeast as a therapeutic strategy for *C. albicans* and other opportunistic pathogens in the reproductive tract (Ling *et al.* 2023).

Finally, the basic science surrounding the role of the gut microbiome in reproductive disease is leading to the development of live bacterial therapeutics. One study showed elevated *B. vulgatus* in females with PCOS, and in murine models, it was linked to a bile acid – IL22 axis as a potential mediator of PCOS pathology. This suggests that genetically engineering a bile acid-modifying or IL22-expressing engineered bacteria could be effective for the treatment of PCOS (Qi *et al.* 2019).

The development of live therapeutics is a rapidly growing field, with an increasing number of scientists recognizing the need for the fusion of synthetic biology, clinical medicine, and basic science. As the field grows, it will hopefully closely integrate these three areas to provide optimal and personalized therapies.

## Conclusion and future directions

Manipulating the microbiome to enhance gynecologic health holds vast, untapped potential. It opens up exciting possibilities for restoring microbial equilibrium and preventing microbiome-associated conditions. Although more work is needed for clinical care recommendations, these studies highlight the potential impact of such strategies in preventing and treating major contributors to female infertility and gynecological maladies, such as endometriosis, PCOS, and BV. One exciting avenue includes leveraging the microbiome to improve pharmaceutical efficacy. Notably, the composition of the vaginal microbiome has been found to impact the effectiveness of tenofovir, an HIV treatment drug, in females (Klatt *et al.* 2017). Furthermore, the advancements and strains of interest mentioned in the probiotics section demonstrate the feasibility of further development of engineered probiotics for therapeutic purposes. While many live bacterial therapeutics are still under development and have been studied *in vivo* or in murine models, their effectiveness in preclinical or early clinical trials is yet to be established. An innovative technology known as the vagina-on-a-chip has recently been developed to replicate the vaginal epithelial microenvironment and its interactions with the microbiome, enabling preclinical validation. This microfluidic culture model of vaginal mucosa can be used to assess colonization, characterize interactions between engineered probiotics and host vaginal epithelium, measure host innate immune response, and test the safety and efficacy of live bacterial therapeutics under development (Mahajan *et al.* 2022).

Despite these advances, progress in this area has been hindered by historical research gaps and gender disparities (Mirin 2021). Until the NIH Revitalization Act of 1993, clinical trials primarily focused on male subjects and often excluded females. Furthermore, the prevailing gender disparities extend beyond clinical trials because venture capitalists, who are predominantly men, have a limited understanding of the market for female technologies (Mann 2022). The historical deficiency in funding and underrepresentation of females in clinical trials has significantly impacted our comprehension of female-dominant conditions. This, coupled with systemic gender biases in medical education and training, has led to many women feeling unheard in healthcare settings, with their pain often disregarded and treatment options primarily focused on symptom management rather than curative approaches (Ferrero *et al*. 2018, Hoeger *et al*. 2021).

Furthermore, few studies have interrogated the microbial community composition within the genital tracts of gender-diverse individuals, representing a key knowledge gap. A complete review of this literature is described by Krakowsky *et al.* (2022). In brief, transgender individuals experiencing gender dysphoria as a result of gender incongruence can elect to undergo gender-affirming care, which alleviates gender dysphoria through increasing congruence of external and internal gender identity. This can be achieved through elective surgeries such as phalloplasties, neovaginoplasties, and/or orchiectomies. Transgender women who have undergone neovaginoplasty have been observed to develop a neovaginal microbiome with an abundance of *Lactobacillus* species present (Petricevic *et al.* 2014). Gender-affirming care may also include hormone replacement therapy with testosterone or estradiol. Both of these sex hormones indirectly influence the vaginal microbiome through changes in epithelial integrity (Baldassarre *et al.* 2013*a*,*b*), and gut microbes influence the levels of circulating sex hormones through microbial estrogen-deconjugating genes (Baker *et al*. 2017) and microbial–testosterone interactions (Li *et al.* 2022), understanding the impacts of gender-affirming therapy on vaginal and neovaginal microbiomes of transgender individuals is critical to accurately providing healthcare. The vaginal microbiome of transgender men undergoing testosterone is understudied – a single 2019 study reports non-*Lactobacillus* dominance and high microbial diversity in the transman vaginal environment (Winston McPherson *et al.* 2019) More work must be done in this area using studies with larger sample sizes to provide equitable microbial healthcare to patients across the gender spectrum.

Another knowledge gap is the understanding of temporal dynamics within the female gut and vaginal microbiome. There are clear circadian dynamics within host–microbe relationships; however, much of the research investigating these diurnal rhythms focuses on only male gut microbiota (Frazier & Leone 2022). There is some consensus that vaginal microbiome composition fluctuates with the menstrual cycle phase; however, further evidence is needed regarding the links to gut microbiota with cycle phase, and the role of hormonally based contraception (Song *et al.* 2020, Krog *et al.* 2022). Genetically engineered mice that express human genes related to menstrual cycle regulation and attempt to mimic hormonal patterning have been developed. However, this model lacks physiological menstruation, and therefore cannot be used to study the vaginal environmental shift that occurs during menstruation (Liu *et al.* 2020). As a result, there is a need for more human studies that analyze the influence of menstrual bleeding on the vaginal microbiome.

This review has highlighted the potential of microbiome manipulation to improve female reproductive health. However, this field is still in its infancy, and much more research and funding is needed to fully understand the role the microbiome plays in female gynecological conditions so that we can wield its power. In writing this, we seek to encourage further research in this promising field and emphasize the importance of exploring microbiome-based interventions in the realm of gynecologic health.

## Declaration of interest

RK is associated with Gencirq (stock and SAB member), DayTwo (consultant and SAB member), Cybele (stock and SAB member), Biomesense (stock, consultant, SAB member), Micronoma (stock, SAB member, co-founder), and Biota (stock, co-founder). JAG is associated with Holobiome (stock, SAB member); BiomeSense (stock, SAB, co-founder); and SunGenomics (stock, SAB member). LB discloses editorial stipends from JAMA, Urogynecology and Up to Date. AZ is a co-founder, acting chief medical officer, and equity holder in Endure Biotherapeutics.

## Funding

AZ is supported by the VA Merit BLR&D Award I01 BX005707, and NIH R01 HL148801, R01 EB030134, R01 AI163483, and U01 CA265719. EM is supported by NIH 5F31HD106762-02. TK is supported by 5T32GM007198-49. MD is supported by 1R34NS126030-01. VT is supported by NIH R01HD095412. Some UC San Diego authors received institutional support from NIH P30 DK120515, P30 DK063491, and UL1 TR001442. The funders had no role in the study design, data collection and interpretation, or the decision to submit the work for publication. The contents do not represent the views of the U.S. Department of Veterans Affairshttp://dx.doi.org/10.13039/100000738 or the U.S. Government.
